# Application-aware deadline constraint job scheduling mechanism on large-scale computational grid

**DOI:** 10.1371/journal.pone.0207596

**Published:** 2018-11-20

**Authors:** Xiaoyong Tang, Xiaoyi Liao

**Affiliations:** 1 College of Information Science and Technology / Southern Regional Collaborative Innovation Center for Grain and Oil Crops in China, Hunan Agricultural University, Changsha, China; 2 School of Information Science and Engineering, Hunan University, Changsha, China; Chongqing Jiaotong University, CHINA

## Abstract

Recently, computational Grids have proven to be a good solution for processing large-scale, computation intensive problems. However, the heterogeneity, dynamics of resources and diversity of applications requirements have always been important factors affecting their performance. In response to these challenges, this work first builds a Grid job scheduling architecture that can dynamically monitor Grid computing center resources and make corresponding scheduling decisions. Second, a Grid job model is proposed to describe the application requirements. Third, this paper studies the characteristics of commercial interconnection networks used in Grids and forecast job transmission time. Fourth, this paper proposes an application-aware job scheduling mechanism (AJSM) that includes periodic scheduling flow and a heuristic application-aware deadline constraint job scheduling algorithm. The rigorous performance evaluation results clearly demonstrate that the proposed application-aware job scheduling mechanism can successful schedule more Grid jobs than the existing algorithms. For successful scheduled jobs, our proposed AJSM method is the best algorithm for job average processing time and makespan.

## Introduction

Computational Grids are a platform that can share, select, and aggregate geographically distributed heterogeneous idle computing resources to achieve vast computation and storage capabilities [1]. In recent years, Grid techniques have been widely used in solving computation intensive problems in physics, genetics, astronomy, civil engineering, and among others [2, 3]. The China National Grid, which consists of large Supercomputing centers, provinces or university computing nodes, is one example of such a computational Grid examples [4]. Many large-scale computation intensive jobs, such as rice genome-wide association analysis, community earth system models, and large airliner CFD (Computational Fluid Dynamics) checks and auxiliary design, have utilized this Grid. To achieve the promised high computing performance, effective and efficient job scheduling mechanisms are fundamentally important in the Grid environment [3, 5].

Grid job scheduling mechanisms aim to effectively exploit the benefits of Grids’ idle computational resources by mapping jobs to appropriate Grid computing centers. This is a well-known NP-complete problem, in the general case, that can exhibit a huge search space of possible scheduling solutions [6–8]. The problem increases in complexity when the Grid computational resources are heterogeneous, dynamic, and even the load on Grid computing centers varies with time.

Most classical Grid job scheduling strategies, including immediate mode and batch mode, are based on the assumption that the Grid resources provided by Grid computing centers are constant in a relatively long period [5, 6]. However, for some actual Grid systems, such as the China National Grid, resources are seriously affected by the Grid computing center’s local systems and Grid systems, and the number of resources changes dynamically [9]. For example, the number of computational nodes provided to the China National Grid by the Changsha National Supercomputing Center may go from 254 to 120 in space of a few minutes.

On the other hand, Grid resources are heterogeneous not only in terms of hardware, such as multicore and manycore type, speed, network capacity, storage, and more, but also in terms of software type, license, version, and so on [10]. The Grid applications also have diverse resource requirements [11–13]. For example, automotive crash simulation analysis needs multidisciplinary finite element solver RADIOSS and pre-processing software HyperMesh, and CPU+GPU coordinated parallel computing [11, 12]. These application requirements are not provided by all Grid computing centers. Furthermore, the Grid computing center’s corresponding software license and number of available computing nodes may not meet application requirements. Some Grid computing centers choose not to support certain specific applications because of security issues, performance impact, or business strategies. Additionally, Grid application data transmission from the job submission point to the Grid computing center is a major challenge. This is owing to the fact that most Grid systems are connected by commercial interconnect networks, the communication bandwidth of which is highly affected by the environments. These application-aware issues are worth further investigation for the job scheduling mechanism.

Motivated by these challenges, this paper designs and evaluates an application- aware job scheduling mechanism (AJSM) for Grids. The major contributions of this work are multifold and can be summarized as follows:

- First, this paper constructs a Grid job scheduling architecture, including a *Job Queue*, *Scheduler*, *Grid Resources Monitor*, *Network Prediction*, *Job Dispatch*, and *Grid environments*, which can dynamically obtain Grid computing centers’ idle resources and make job scheduling decisions.
- Secondly, this paper studies the communication characteristics of Grid computing centers based on commercial interconnection networks, and adopt an ARIMA model to forecast data transfer bandwidth and job transmission time.
- Thirdly, this paper builds a Grid job model to accommodate application requirements, and normalize the heterogeneous Grid computing resources as a standard multicore and manycore computational node model. This paper also formulates the application-aware deadline constraint job scheduling problem as a linear programming problem.
- Fourthly, this paper proposes an application-aware job scheduling mechanism (AJSM), which mainly consists of periodic scheduling flow and a heuristic job scheduling algorithm. The heuristic scheduling algorithm first tries to find Grid computing centers that can satisfy jobs software requirements. Then, the ARIMA job transmission time is applied, and the algorithm schedules job to corresponding Grid computing center.
- Finally, performance evaluation are conducted and the experimental results show that our proposed AJSM algorithm can successful schedule more Grid jobs than MGA, Min-Min. The AJSM method also outperforms existing algorithms in terms of job average processing time and makespan.

The rest of the paper is organized as follows: related works are summarized in Section 2. The computational Grid model, job scheduling architecture, and job model are described in Section 3. In Section 4, this paper provides a Grid job transmission time prediction method based on an ARIMA model. The paper presents Grid heterogeneous computing nodes, scheduling attributes, and problems in Section 5. Section 6 proposes an application-aware job scheduling mechanism. Performance evaluation is given in Section 7, where the performance of AJSM is assessed in comparison with two similar algorithms. Finally, this paper summarize the contributions and comment on the future directions of this work in Section 8.

## Related works

Many effective heuristic and meta-heuristic Grid scheduling algorithms have been proposed to obtain near-optimal solutions, such as MET (Minimum Execution Time), Min-Min, Max-Min, and XSufferage [6, 14]. The Min-Min heuristic algorithm tries to schedule job with overall minimum execution finish time. In contrast to Min-Min, the heuristic Max-Min algorithm chooses the job and Grid center pair with the maximum minimum execution finish time. Min-Min and Max-Min have been extended to adapt to different Grid job scheduling solutions. For example, Vaaheedha and Nazreen proposed a MiM-MaM algorithm, which combines Min-Min and Max-Min to overcome their drawbacks [15].

Bioinspired meta-heuristic algorithms are another class of scheduling mechanisms applied effectively to Grid [7, 16–20]. Liu *et al.* extended conventional particle swarm optimization particles’ positions and velocities from real vectors to fuzzy matrices. This scheduling method can dynamically generate an optimal schedule solution [21]. In work [19], the authors applied an automatically controlled ant colony optimization (ACO) method to Grid job scheduling, which effectively processes the effect of interprocess communication and optimizes the turnaround time of the job. Tiwari and Vidyarthi introduced lazy ants into the Grid job scheduling ACO and obtained a good balance between diversification and convergence of the search process [7]. This algorithm not only produces a good solution for the given objectives but also reduces the time complexity of the algorithm. In paper [16], the authors enhanced a genetic algorithm’s main branching operations, and implemented a Grid job scheduling method which can simultaneously optimize two objectives: makespan and flowtime. Considering the Grid resources availability, Prakash and Vidyarthi proposed a Grid scheduling technique based on a genetic algorithm [17]. Younis and Yang proposed an improved genetic algorithm (MGA) that adopts a new mutation procedure to solve grid independent job scheduling problem [18]. Tang *et al.* proposed a hybrid algorithm combining Genetic Algorithm and Simulated Annealing Algorithm to search optimal solution in designing reasonable departure schedule [22]. However, these scheduling strategies can not effectively deal with Grid application requirements.

The application-aware Grid job scheduling problem reported in the literature was proposed by Hu and Veeravalli [10], whose RAPAR and RAKAR algorithms addressed the scheduling of applications with heterogeneous processing requirements on a Grid. Paper [23] considered the geographically distributed data feature of Big Data applications and proposed an advance reservation scheduling framework in optical Grid. Xu and Yang proposed a heuristic multi-objective scheduling algorithm to optimize both Grid users’ applications and Grid resource providers’ incentives, such as cost [24]. Other heuristic scheduling optimization techniques are conventional k-means cluster scheduling [25], cost-driven partial critical paths scheduling [26], dynamic programming [27, 28], K-Percent Best(KPA) [29], rank-based hybrid scheduling (RBHS) [30]. This paper will consider the hardware, software, and job transmission time of Grid application requirements, and make optimization scheduling decisions to improve computational Grid performance.

## Computational grid and job models

This section describes the target computational Grid, job scheduling architecture, and job model used in our study.

### Large-scale computational Grid

**Fig 1:**
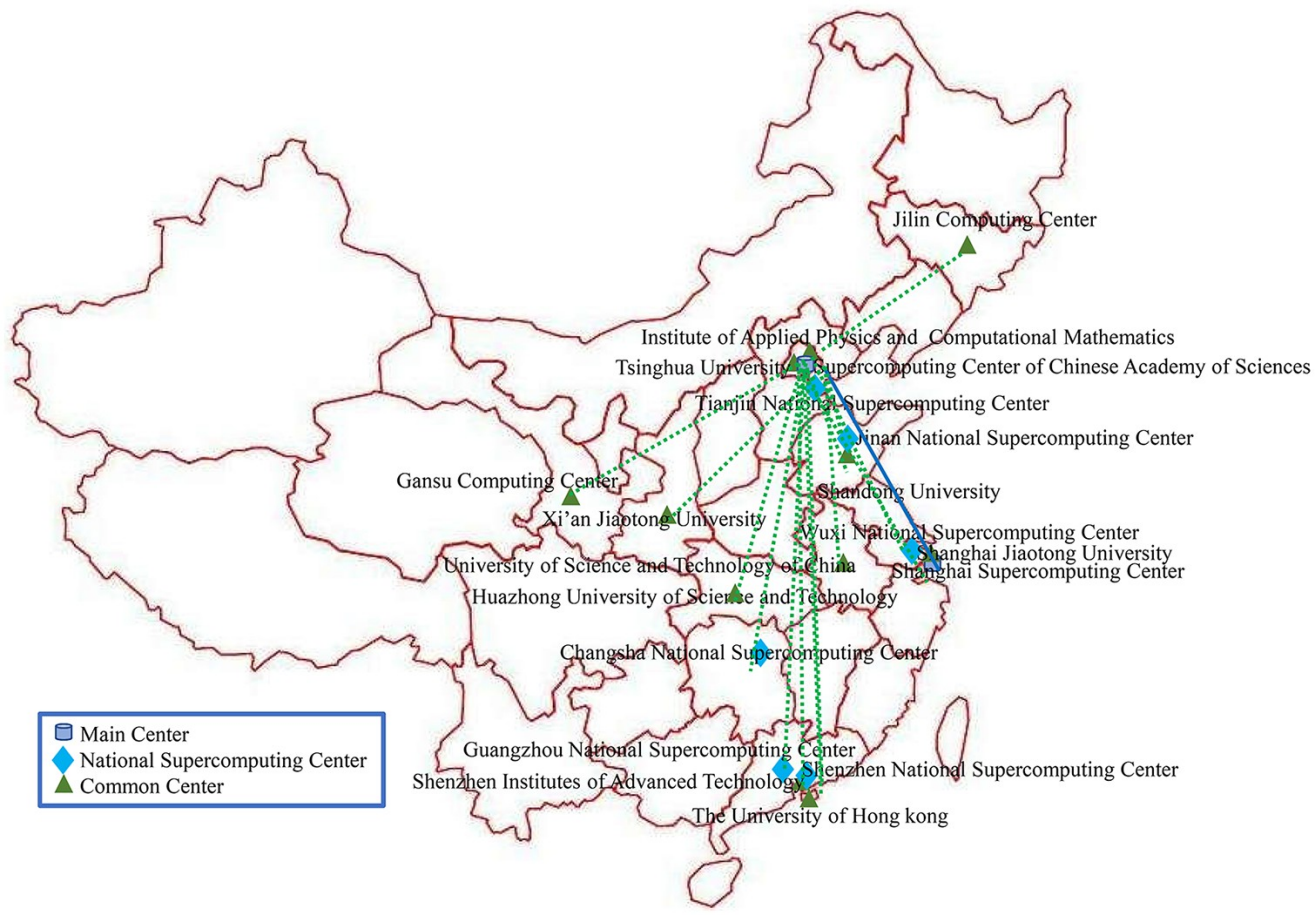
China National Grid.

This paper studies the China National Grid, which consists of many geographically distributed heterogeneous computing resources, including 2 main centers, 6 National Supercomputing centers, and 11 common centers (Fig 1). The Supercomputing center of the Chinese Academy of Sciences is one of the main centers and is responsible for managing the whole Grid. The National Supercomputing centers are Wuxi, Changsha, Jinan, Guangzhou, Shenzhen, and Tianjin. All of the National Supercomputing centers have powerful computing capability, with resources such as the Sunway TaihuLight and Tianhe-2, the top 2 supercomputers in a recent TOP500 list [31]. The centers of the Grid are connected by ChinaNet or CerNet, which have heterogeneous public commercial interconnection bandwidth and delay.

Each Grid computing center *GC*_*i*_ provides many parallel computational software packages, such as Molecular Massively Parallel Simulator(LAMMPS), CPMD, GAMES, MPI, SANSYS, RADIOSS, HyperMesh, and so on. This paper uses *PS*(*GC*_*i*_) to denote the set of available software. Each software has attributes: software name, software id, license, and version. The symbols *TN*(*GC*_*i*_), *AN*(*GC*_*i*_), and *AS*(*GC*_*i*_) denote the total number of computational nodes, available computational nodes, and available computational storage of the Grid computing center *GC*_*i*_, respectively. This paper uses the symbol *MM*(*GC*_*i*_) to indicate that the computational node can work as a multicore and manycore model.

### Job scheduling architecture

**Fig 2:**
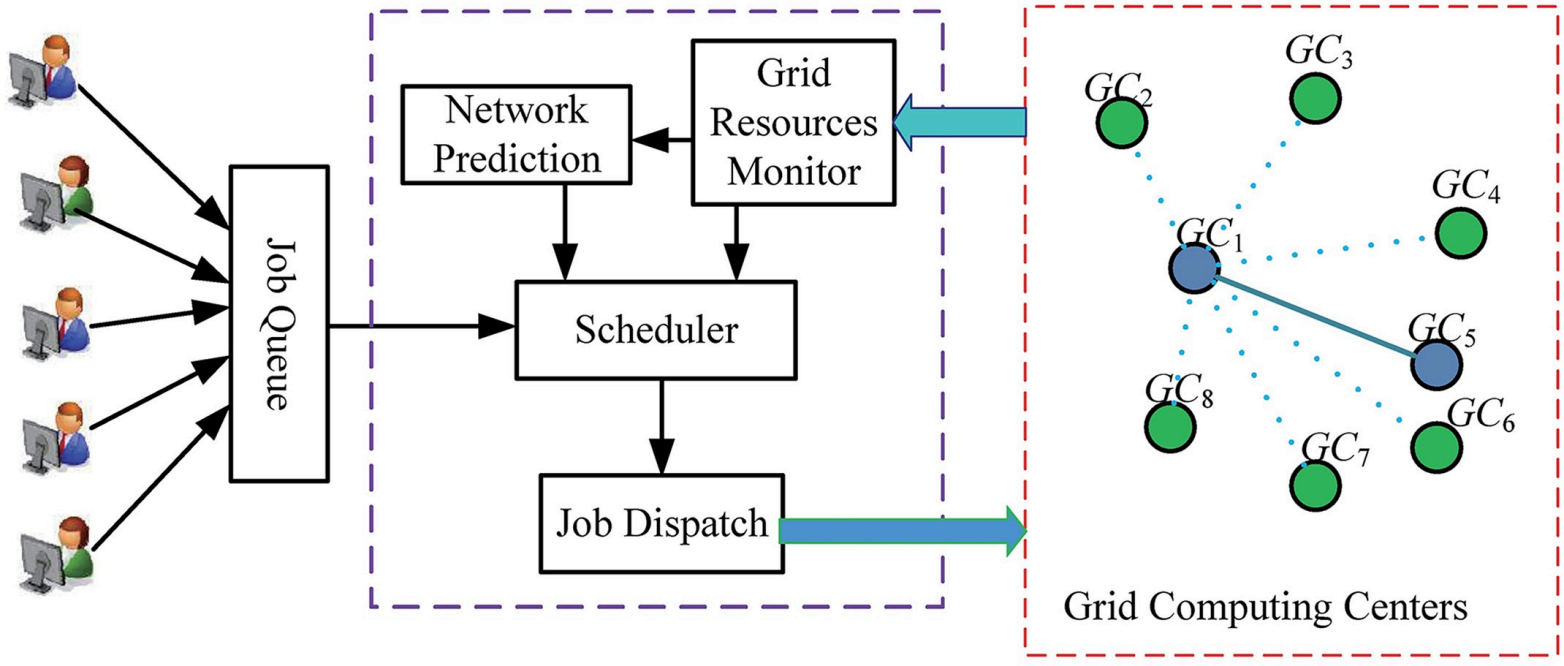
The Grid scheduling architecture.

Fig 2 depicts the large-scale computational Grid job scheduling architecture. This architecture assumes that all applications or jobs, along with their software, computing nodes, execution time, deadlines, storage, and so on, provided by user, are submitted to the main center by a web interaction interface. All jobs are inserted into the job linked list queue and can be periodically scheduled by the *Scheduler*, which is a scheduling decision module, according to the requirements of the application, Grid network prediction, and the dynamic Grid environments. The module *Grid Resources Monitor* can periodically collect Grid computing centers running jobs, available computing nodes, cores, storage, network bandwidth, delay, and so on. The resources of Grid computing centers change dynamically with local and Grid job assignment, job operation completion, resource failure, and safety maintenance. Therefore, in the scheduling architecture, Grid computing centers will report their resource status to the main Grid center at an interval of 4 minutes. *Network Prediction* is used to dynamically forecast future network communication conditions among the main center and other Grid computing centers. *Job Dispatch* can dispatch jobs to the corresponding computing center according to scheduling decisions.

### Grid application model

This paper only considers the scheduling of bag-of-tasks (BoT) or parameter-sweep applications (jobs) on a large-scale distributed computational Grid. Therefore, the jobs *A*_1_, *A*_2_, ⋯, *A*_*N*_ are assumed to be independent and atomic. Examples of these Grid jobs include Monte Carlo simulations [32], tomographic reconstructions, rice genome-wide association analysis [33], and data mining algorithms. They are frequently used in fields such as astronomy, bioinformatics, high energy physics, and many others.

In our application model, each job *A*_*i*_ has requirements, such as, software (including version and license), number of computational nodes, manycore demand, and so on. Furthermore, the job also has characteristics of size, arrival time, execution time, deadline, and more. The Grid application notations and their meanings used throughout this paper are listed in Table 1.

**Table 1:** Grid job *A*_*i*_ characteristics.

*Sw*(*A*_*i*_)	Job executing software
*Sv*(*A*_*i*_)	Software version
*Sl*(*A*_*i*_)	Software license
*Jn*(*A*_*i*_)	Computational nodes
*Jm*(*A*_*i*_)	Manycore demand
*Si*(*A*_*i*_)	The size of whole job
*At*(*A*_*i*_)	The job arrival time
*Et*(*A*_*i*_)	The job execution time
*Dl*(*A*_*i*_)	The job deadline

## Grid job transmission

### Grid data transfer characteristics

**Fig 3:**
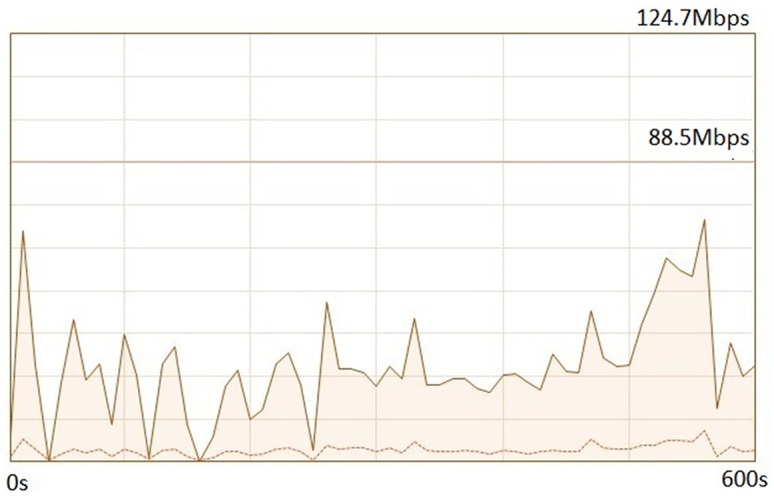
A data transfer bandwidth variance curve.

The performance of data transfer between the main Grid computing center *GC*_1_ and other Grid computing centers *GC*_*i*_ changes with time. This is owing to the fact that most Grids, such as the China National Grid, are interconnected by multi-commercialized internet and not by a dedicated interconnected network. For example, the Changsha National Supercomputing Grid center has China Telecom and China Unicom Internet as its ISPs, and the quality of service from each is different to the other. Another reason is that the commercialized internet is greatly affected by the network environment. Therefore, data transfer bandwidths vary with time. Fig 3 shows a data transfer bandwidth variance curve between the China National Grid main Grid computing center (Supercomputing Center of Chinese Academy of Sciences) and the Changsha National Supercomputing Grid center.

From Fig 3, we can conclude that the data-transfer bandwidth is a set of values of a variable during a consecutive time series. This non-stationary time series can be forecasted by many existing prediction techniques, such as ARIMA model [34, 35], Hidden Markov Model [36], auto-regressive [37], and so on. G. Zhang *et al.* proved that the ARIMA is one of most suitable prediction models for server workload, resource, and communication network with high efficiency and low time complexity [35]. Therefore, this paper uses ARIMA model to forecast Grid job data transmission time among Grid computing centers.

### Job transmission time prediction

The ARIMA model is the combination of Auto Regressive (AR) and Moving Average (MA) models, and was developed by Box and Jenkins [34]. Generally, ARIMA is model as *ARIMA*(*p*, *d*, *q*), which has the following concise form

$$\begin{array}{c} \phi_{p}\left(B\right)\nabla^{d}x_{t}=\theta_{q}\left(B\right)e_{t}. \end{array}$$ (1)

where *x*_*t*_ is the prediction of Grid data-transfer bandwidth at time *t*, **B** is the backward shift operator, *ϕ*_*p*_(**B**) is the Auto Regressive operator defined as *ϕ*_*p*_(**B**) = 1 − *ϕ*_1_**B** − *ϕ*_2_**B**^2^ − ⋯ − *ϕ*_*p*_**B**^*p*^, and ∇^*d*^ = (1 − **B**)^*d*^ is the *d*th order of difference operator. *e*_*t*_ is the normally distributed error at period *t*, *θ*_*q*_(**B**) = 1 − *θ*_1_**B** − *θ*_2_**B**^2^ − ⋯ − *θ*_*q*_**B**^*q*^. The ARIMA model uses previous time series data-transfer bandwidths *x*_*t*−1_, *x*_*t*−2_, ⋯ to forecast *x*_*t*_.

In this paper, the time period is set as 5*s*. Therefore, at time period *t*, the Grid can transfer 5*x*_*t*_M data from the main Grid center to the corresponding computing center. The data-transfer bandwidth *x*_*t*_ also can be iteratively used to forecast the next time series *x*_*t*+1_, *x*_*t*+2_, *x*_*t*+3_, ⋯. Thus, for Grid job *A*_*i*_, the data transmission prediction time *DPT*(*A*_*i*_, *GC*_*k*_) from the main Grid center to the Grid computing center *GC*_*k*_ can be expressed as

$$\begin{array}{c} \left\{ \begin{array}{c} Si\left(A_{i}\right)=5\sum x_{t+s},\;\;\;\;s\in 0,1,\cdots ,r,\\ DPT\left(A_{i},GC_{k}\right)=5\times \left(r+1\right). \end{array}\right. \end{array}$$ (2)

where *r* is the max data transfer periods.

## Problem formulation

### Heterogeneous computational node normalization

The computation capacity of Grid computing centers is naturally heterogeneous. For example, the Tianhe-2 supercomputer in the Guangzhou National Supercomputing Center has 17920 computational nodes, each node has 2 Intel Xeon E5-2692v2 12C 2.2GHz processors and 3 Xeon Phi 57 [38]. The Dawning Nebulae supercomputer in the Shenzhen National Supercomputing Center has 2560 computational nodes, each node has 2 Intel Xeon 6C 2.66GHz processors and 1 NVidia C2050 GPU [39]. Therefore, an important task for job scheduling is to standardize the heterogeneous Grid computing center computation capacity.

There are many research work to address heterogeneity from engineering disciplines. Zou *et al.* applied a generalized finite mixture of negative binomial (NB) models with K mixture components to solve heterogeneous data in empirical Bayes estimation [40]. Fan *et al.* use deep learning method to virtualize heterogeneous radio into normalized resources [41]. These methods are very effective for solving the corresponding problems, but they are not suitable for our proposed periodic scheduling mechanism because of their high time complexity. In the following, we propose a simple and efficient heterogeneous computational node normalization method.

In this paper, we adopt 2 CPUs, which have 6 cores at 2.0GHz, as the computational node standardization multicore capacity. Here, systems let *GMS*(*GC*_*i*_) and *GMC*(*GC*_*i*_) denote the speed and cores of the Grid computing center *GC*_*i*_ CPU, respectively. *GMN*(*GC*_*i*_) is the CPU number of the Grid computing center *GC*_*i*_ computational node. Therefore, the standardization multicore capacity *GSC*(*GC*_*i*_) of the Grid computing center *GC*_*i*_ computational node is

$$\begin{array}{c} GSC\left(GC_{i}\right)=\frac{GMN\left(GC_{i}\right)\times GMC\left(GC_{i}\right)\times GMS\left(GC_{i}\right)}{2\times 6\times 2.0}. \end{array}$$ (3)

For the computational node manycore capacity, this paper adopts the NVIDIA Tesla C2050, which has 448 cores and a computational capacity of 515.0*GFlops*, as the standardization capacity. The single core capacity among manycores, such as NVIDIA, Xeon Phi, SW26010, and so on, is heterogeneous. Therefore, this paper gives a heterogeneity *ϕ* for manycores other than NVIDIA. For example, the manycore heterogeneity *ϕ* of Xeon Phi to NVIDIA is *ϕ* = 2.3. Here, this paper also defines *MCC*(*GC*_*i*_) as the manycore computational capacity of Grid computing center *GC*_*i*_. The computational node standardization manycore capacity *MSC*(*GC*_*i*_) is defined as

$$\begin{array}{c} MSC\left(GC_{i}\right)=\frac{\phi \times MCC(GC_{i})}{515.0}. \end{array}$$ (4)

### Scheduling attributes

To facilitate the presentation of the proposed application-aware constraint job scheduling algorithm, it is necessary to introduce some definitions and assumptions. Let *ET*(*A*_*i*_, *GC*_*k*_) denote the execution time of job *A*_*i*_ on Grid computing center *GC*_*k*_, such that:

$$ET\left(A_{i},GC_{k}\right)=\{\begin{array}{ll} \frac{Et\left(A_{i}\right)}{MIN\left\{GSC\left(GC_{k}\right),MSC\left(GC_{k}\right)\right\}} & Jm\left(A_{i}\right)\;is\;true\\ \frac{Et\left(A_{i}\right)}{GSC\left(GC_{k}\right)} & Otherwise. \end{array}$$ (5)

where *ET*(*A*_*i*_, *GC*_*k*_) is the maximum execution time between multicore and manycore processors on a computational node when the application manycore requirement *Jm*(*A*_*i*_) is true. Otherwise, the application *A*_*i*_ only uses the multicore of the computational node. The job *A*_*i*_ execution finish time *JFT*(*A*_*i*_, *GC*_*k*_) on Grid computing center *GC*_*k*_ is the sum of the scheduling point, job transmission prediction time, and job execution time, and can be defined as follows

$$\begin{array}{c} JFT\left(A_{i},GC_{k}\right)=sPoint_{j}+DPT\left(A_{i},GC_{k}\right)+ET\left(A_{i},GC_{k}\right). \end{array}$$ (6)

where *sPoint*_*j*_ is the system periodic scheduling point with interval 120*s* (2 minutes according to scheduling architecture module *Grid Resources Monitor*). In fact, the system periodic scheduling point *sPoint*_*j*_ is the current scheduling time, such as 13: 47: 12, and the next scheduling point *sPoint*_*j*+1_ will be 13: 49: 12. Thus, job *A*_*i*_’s actual processing time *JPT*(*A*_*i*_, *GC*_*k*_) is the difference between its execution finish time and arrival time. This paper expresses it as

$$\begin{array}{c} JPT\left(A_{i},GC_{k}\right)=JFT\left(A_{i},GC_{k}\right)-At\left(A_{i}\right). \end{array}$$ (7)

On the contrary, the job scheduling strategies are constrained by application software and hardware requirements. Each Grid computing center provides an application software set *PS*(*GC*_*i*_), and the software license and version must satisfy the job requirements. That is to say that the license *li*(*sf*_*k*_) and version *vs*(*sf*_*k*_) for application software *sf*_*k*_ ∈ *PS*(*GC*_*i*_) must be higher than job *A*_*i*_’s software *Sw*(*A*_*i*_) requirements: license *Sl*(*A*_*i*_) and version *Sv*(*A*_*i*_). That is,

$$\begin{array}{c} \left\{ \begin{array}{c} Sw\left(A_{i}\right)=sf_{k}\;\wedge \;sf_{k}\in PS\left(GC_{i}\right),\\ li\left(sf_{k}\right)\geq Sl\left(A_{i}\right),\\ vs\left(sf_{k}\right)\geq Sv\left(A_{i}\right). \end{array}\right. \end{array}$$ (8)

The Grid computing center *GC*_*k*_ must satisfy job *A*_*i*_ hardware requirements, such as manycore support, available computational nodes, and available storage and can be expressed as

$$\begin{array}{c} \left\{ \begin{array}{c} Jn\left(A_{i}\right)\leq AN\left(GC_{k}\right),\\ Js\left(A_{i}\right)\leq AS\left(GC_{k}\right),\\ MM\left(GC_{i}\right)=true\;\;\;\;if\;Jm\left(A_{i}\right)\;is\;true. \end{array}\right. \end{array}$$ (9)

Generally, jobs are also expected to be completed before their deadline. That is,

$$\begin{array}{c} JFT\left(A_{i},GC_{k}\right)\leq Dl\left(A_{i}\right). \end{array}$$ (10)

### Problem statement

This section sets *X*_*i*_ = 1 if job *A*_*i*_ is scheduled on Grid computing center *GC*_*k*_, and *X*_*i*_ = 0 if job *A*_*i*_ is rejected by the system and the Grid system can not find a suitable Grid computing center *GC*_*k*_ to accomplish its execution. Therefore, the total processing time of jobs *TPT* can be expressed as

$$\begin{array}{c} TPT=\sum_{i=1}^{m}X_{i}JFT\left(A_{i},GC_{k}\right). \end{array}$$ (11)

Here, this paper outlines the main scheduling objectives used in this study. The first performance objective is the average processing time *APT*, which is the average of all jobs actual processing time and is defined as

$$\begin{array}{c} APT=\frac{\sum_{i=1}^{m}X_{i}JFT\left(A_{i},GC_{k}\right)}{\sum_{i=1}^{m}X_{i}}. \end{array}$$ (12)

where *m* is the total number of jobs in the Grid system including many scheduling point jobs. The other scheduling objective is to try to degrade the job rejection ratio *JobRej*, which is defined as

$$\begin{array}{c} JobRej=\frac{m-\sum_{i=1}^{m}X_{i}}{m}100\%. \end{array}$$ (13)

This paper tries to minimize both the average processing time and job rejection ratio. This optimization scheduling problem can be expressed as

$$\begin{array}{c} \left\{ \begin{array}{c} Minimize\;APT\;and\;JobRej,\\ Subject\;to\;\;\;\;Eq(8),Eq(9),Eq(10). \end{array}\right. \end{array}$$ (14)

## Application-aware job scheduling mechanism

The proposed application-aware job scheduling mechanism (AJSM) tries to periodically schedule jobs by using an application-aware deadline constraint job scheduling algorithm. The following sub sections will describe the main ideas.

### The periodic scheduling flow

**Fig 4:**
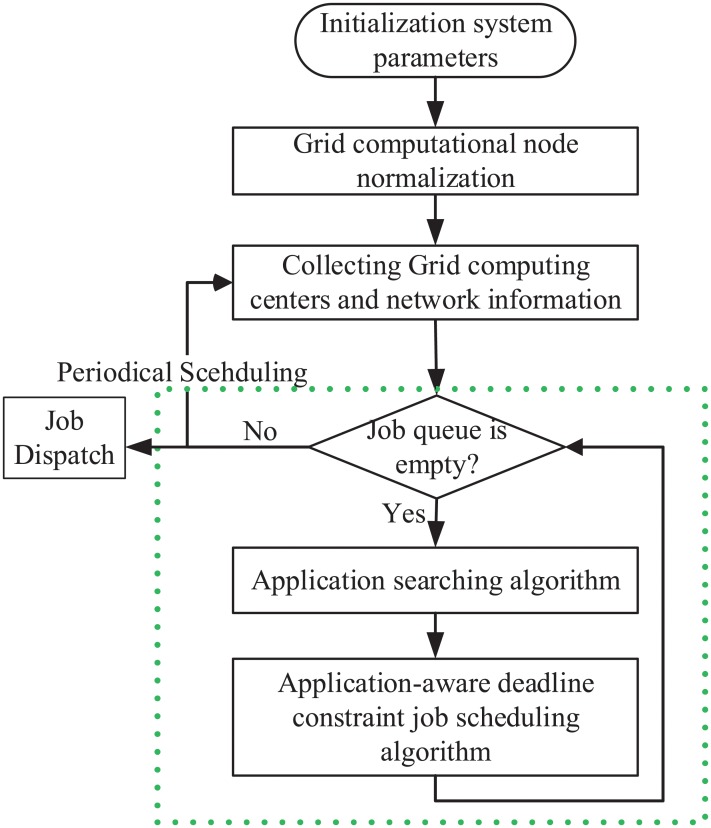
The application-aware periodic job scheduling flow.

This section proposes an application-aware periodical scheduling flow, as shown in Fig 4. The Grid job scheduling mechanism first initializes system parameters, such as the scheduling point *periodSch* = 0, the Grid computing centers’ software, the total number of computational nodes *TN*(*GC*_*i*_), and so on. The Grid computing centers’ heterogeneous computational nodes are then normalized according to Section heterogeneous computational node normalization. Next, the Network Prediction and Grid Resources Monitor module are adopted to periodically collect Grid computing centers and network information, which are used in the later scheduling decision. The interval of periodic scheduling is set to 4 minutes according to the Grid Resources Monitor module. Lastly, the application-aware deadline constraint job scheduling algorithm is responsible for scheduling all jobs submitted by users in each period.

### Application-aware deadline constraint job scheduling algorithm

Our proposed application-aware deadline constraint job scheduling algorithm first needs to find Grid computing centers that can satisfy job (or application) software requirements. This process is outlined in Algorithm 1, which attempts to find the set of available Grid computing centers *Avc*(*A*_*i*_) for each job. The set *Avc*(*A*_*i*_) must satisfy Eq (8) to accommodate the job software requirements. The algorithm rejects job *A*_*i*_ only if the available Grid computing centers set *Avc*(*A*_*i*_) is empty.

**Algorithm 1:** Grid computing centers search algorithm.

**Input:** Grid computing centers’ application software set *PS*(*GC*_*i*_) and Grid jobs.

**Output:** The job available Grid computing center set *Avc*(*A*_*i*_).

1 **for** *each Grid job A*_*i*_ **do**

2  **for** *each Grid computing center GC*_*i*_ **do**

3   **for** *each application software sf*_*k*_ ∈ *PS*(*GC*_*i*_) **do**

4    **if** Eq 8 *is true* **then**

5     Put *GC*_*i*_ into job *A*_*i*_’s available Grid computing center set

     *Avc*(*A*_*i*_).

6    **end**

7   **end**

8  **end**

9  Remove job *A*_*i*_ from Grid job set.

10  **if** *Avc*(*A*_*i*_) *is empty* **then**

11   Reject job *A*_*i*_.

12  **end**

13 **end**

The application-aware deadline constraint job scheduling algorithm is formalized in Algorithm 2. The goal of this algorithm is to the deliver job that has the minimum execution finish time with the application requirements and deadline constraints on the Grid. To achieve this goal, the algorithm first uses the Grid computing centers search algorithm to find the job’s available Grid computing centers *Avc*(*A*_*i*_). Next, for any unscheduled jobs, our proposed algorithm uses the ARIMA forecast transmission time *DPT*(*A*_*i*_, *GC*_*k*_) and computes the job’s minimum execution finish time on its available Grid computing centers (Steps 6-8). If the computing resource demands and the job’s deadline constraint are met, the Grid computing center is put into job’s schedulable set (Steps 9-11). Steps 13-18 try to find a Grid computing center with the minimum execution finish time for job *A*_*i*_. If there is no Grid computing center that can run job *A*_*i*_, job *A*_*i*_ will be inserted into the next scheduling point queue until the system rejects it. Lastly, this algorithm assigns the job to the Grid computing center with minimum *JFT*(*A*_*i*_, *GC*_*k*_) for all job and Grid computing center pairs, and updates Grid resource and job scheduling queue information.

**Algorithm 2:** Application-aware deadline constraint job scheduling algorithm.

**Input:** Grid jobs.

**Output:** An assignment (*A*_*i*_, *GC*_*k*_) of job *A*_*i*_ and Grid computing center *GC*_*k*_.

1 Initialize Grid computing center parameters;

2 Grid computing centers search algorithm;

3 **while** *job queue is not empty* **do**

4  **for** *each unscheduled job A*_*i*_ **do**

5   **for** *each Grid computing center GC*_*k*_ ∈ *Avc*(*A*_*i*_) **do**

6    Compute job execution time *ET*(*A*_*i*_, *GC*_*k*_) (Eq (5));

7    Use ARIMA forecast *DPT*(*A*_*i*_, *GC*_*k*_) (Eq (2));

8    Compute job execution finish time *JFT*(*A*_*i*_, *GC*_*k*_) (Eq (6));

9    **if** Eqs (9) and (10) *are satisfied* **then**

10     Put *GC*_*k*_ into job *A*_*i*_’s schedulable set.

11    **end**

12   **end**

13   **if** *Job A*_*i*_’ *schedulable set is empty* **then**

14    Put job *A*_*i*_ into the next scheduling point

15   **end**

16   **else**

17    Find a GC with minimum *JFT*(*A*_*i*_, *GC*_*k*_) for job *A*_*i*_.

18   **end**

19  **end**

20  Find *A*_*i*_ and *GC*_*k*_ pair with minimum *JFT*(*A*_*i*_, *GC*_*k*_);

21  Assign job *A*_*i*_ to Grid computing center *GC*_*k*_;

22  Update Grid center *AN*(*GC*_*k*_) and *AS*(*GC*_*k*_);

23  Remove job *A*_*i*_ from job queue.

24 **end**

### Time complexity

The time complexity of job scheduling algorithms is usually expressed in terms of the number of jobs *N*, the Grid computing centers *W*, and the maximum number of software packages *Z*. The time complexity of this application-aware deadline constraint job scheduling algorithm is analyzed as follows: The application searching algorithm can be done in time *O*(*NWZ*). In fact, the time complexity of the ARIMA prediction method is much higher than steps 6 and 8-11. Here, this paper assumes that the ARIMA prediction method *O*(*ARIMA*) as the proposed algorithm’s basic time complexity, and the time complexity of steps 4-18 can be done in time *O*(*ARIMA* × *NW*). Therefore, finding the job and Grid computing center pair with minimum *JFT*(*A*_*i*_, *GC*_*k*_) in steps 3-19 can be done in time *O*(*ARIMA* × *N*^2^*W*). Notice that the most time consuming computation is the loop in Step 7. Thus, the overall time complexity of the algorithm is *Max*{*O*(*NWZ*), *O*(*ARIMA* × *N*^2^*W*)}.

## Performance evaluation

To assess the performance of proposed AJSM, this paper developed a discrete-event simulation Grid environment based on GridSim [42]. This paper compares the AJSM algorithm with a baseline traditional scheduling strategies Min-Min [6] and a recently new meta-heuristic algorithm MGA [18] to understand its effectiveness on Grids. The performance metrics chosen for the comparison are the all jobs total processing time *TPT* in Eq (11), average processing time *APT* in Eq (12), makespan, and job rejection ratio *JobRej* in Eq (13). Here, makespan is the maximum job finish time for all jobs and defined as

$$\begin{array}{c} makespan=Max_{i=1,2,\cdots ,m}\left\{X_{i}JFT\left(A_{i},GC_{k}\right)\right\}. \end{array}$$ (15)

The Min-Min algorithm begins with computing the set of minimum completion time for each unmapped Grid jobs (or applications) on all Grid computing centers. Then, the job with the overall minimum completion time is chosen and allocated to the corresponding Grid computing center. Last, the newly mapped job is removed from unmapped Grid job set and the process repeats until all jobs are scheduled. The Min-Min is a traditional and widely used scheduling algorithm that has been adopted by many research works as a reference object or evaluation benchmark [6, 14, 15, 18]. The improved genetic algorithm (MGA) starts with an initial population, which is generated by seeding the population with one individual generated by Min-Min, and the other individuals generated randomly. Then, the following steps: selection, crossover, and mutation operators are applied. The key of this MGA algorithm is that its mutation operator uses the concept of swap and transfer to alter individuals [18]. This is an effectively and newly scheduling strategy that we choose to compare with our proposed mechanism.

### Experimental settings and environments

In the following experiments, this paper simulates 20 Grid computing centers with different characteristics, such as number of computational nodes, application software set, and storage, while each node has multicore (CPU, core, speed), manycore (capacity, heterogeneity *ϕ*), and memory characteristics. The main parameters of the simulated computing resources are listed in Table 2. The first 10 Grid computing centers (*GC*_1_, ⋯, *GC*_10_) are derived from the China National Grid [4], where their total number of nodes is up to 74626. The other 10 Grid computing centers (*GC*_11_, ⋯, *GC*_20_) are small servers with same configuration. Here, the Grid computing center *GC*_1_ is set as the main center, and the network communication among *GC*_1_ and other centers is a dynamical generated uniformly distribution between 100*M* and 50*G*. The available computational nodes are divided into three categories according to their properties; The first is busy, and the number of available computational nodes is randomly generated as [0.5%–3%] of their total nodes, such as *GC*_4_, *GC*_5_, *GC*_8_, *GC*_16_; the second has medium resources available with [3%–10%], such as *GC*_1_, *GC*_2_, *GC*_9_, *GC*_12_; the third has resource availability of [10%–50%].

**Table 2:** The settings of simulated Grid computing center.

Grid Center	Nodes	Storage(TB)	Software	Multicore			Manycore		Memory(GB)
				CPUs	Cores	Speed(GHz)	Capacity(GHz)	*ϕ*	
*GC*_1_	2048	1.47	78	2	6	2.93	140.64	1	48
*GC*_2_	17920	12.4	112	2	12	2.2	188.1	2.3	64
*GC*_3_	2560	0.408	48	2	6	2.66	515	1	24
*GC*_4_	128	1.5	20	4	8	2.4			128
*GC*_5_	40960	230	75				2381.1	1.9	32
*GC*_6_	912	600	132	2	6	2.5			64
*GC*_7_	980	160	44	2	12	2.5			128
*GC*_8_	7168	262	88	2	6	2.93	515	1	32
*GC*_9_	1650	45	26	4	4	2.0			48
*GC*_10_	300	23	46	2	12	2.5			32
*GC*_11_, ⋯, *GC*_20_	1	0.8	10	1	12	3.0			16

In the simulations, the Grid applications (or jobs) and their application software come from the field of natural science and engineering. Examples include automobile frame stiffness analysis, bridge wind characteristics numerical simulation, mesoscale numerical weather forecast, large airliner CFD check and auxiliary design, and more. These jobs characteristics are derived from the Parallel Workloads Archive HPC2N trace [43] and China National Grid real-world applications [4]. Table 3 lists three jobs characteristics as an example. Grid applications submitted by the user vary from 960 to 2880 with 240 steps, and the scheduling periods are set as 60 and 120 (meaning 4 and 8 hours).

**Table 3:** Three jobs characteristics.

Job	Software	Version	License	Computational nodes	Manycore demand	Execution Time(s)	Size	Deadline(s)
*A*_1_	CP2K	4.1	20	45	No	2737	0.8*G*	3000
*A*_2_	NAMD	2.12		1024	Yes	17890	0.3*G*	20000
*A*_3_	CASTEP	16.4		20	No	1751	1.37*G*	2000

### Job transmission prediction results

As job transmission time is an important factor in job execution finish time, this paper will evaluate our prediction method based on the ARIMA model in the first experiments. This paper tests the above applications among the Grid main computing center and other centers. Network communication historical data are retrieved from the China National Grid. Table 4 lists 10 applications transmission prediction time and their actual time. From Table 4, this paper can conclude that our proposed prediction method is effective for 7 jobs, with an error ratio lower than 10% in all 10 applications.

**Table 4:** The experimental results of job transmission time prediction.

Job	Prediction(s)	Actual(s)	Error rate(%)	Job	Prediction(s)	Actual(s)	Error rate(%)
*A*_1_	32.5	28.9	12.4	*A*_2_	212.3	219.6	3.3
*A*_3_	346.9	332.1	4.5	*A*_4_	72.5	108.9	33.4
*A*_5_	34.8	33.9	2.7	*A*_6_	56.7	57.3	1
*A*_7_	873.7	940.8	7.1	*A*_8_	77.8	85.3	8.8
*A*_9_	708.4	755.6	6.2	*A*_10_	347.8	785.6	55.7

### Experimental results

**Fig 5:**
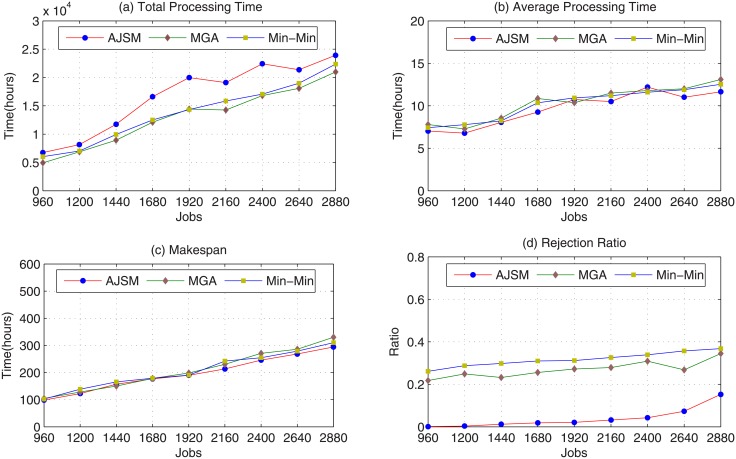
Performance impact of jobs with 60 scheduling points. (a) Total Processing Time; (b) Average Processing Time; (c) Makespan; (d) Job Rejection Ratio.

In the second experiments, this paper first compares the performance of AJSM, MGA, and Min-Min with 60 scheduling periods; the experimental results are shown in Fig 5. From Fig 5(d), this paper can conclude that the job rejection ratio of AJSM is much lower than the other two algorithms. For the average rejection ratio, AJSM significantly outperforms MGA by 85.3%, and Min-Min by 87.5%. This improvement is due to the fact that the AJSM approach is an application-aware algorithm, which can adaptively search Grid computing centers that satisfy jobs software and hardware requirements. Whereas, MGA and Min-Min do not comprehensively consider the computation intensive Grid applications’ requirements, especially for their computing software characteristics. Thus, some jobs scheduled by MGA and Min-Min can not execute on the corresponding Grid computing center and are rejected by the Grid systems, regardless of the existence of other Grid computing centers that can execute those jobs. In contrast, jobs rejected by AJSM are mainly due to the Grid systems lacking a Grid computing center that can meet their software, hardware, and deadline constraints. Therefore, Our proposed algorithm ASJM is more successful than MGA, Min-Min in scheduling Grid jobs.

This paper also observe from Fig 5(d) that as the number of jobs increases, the job rejection ratio of AJSM, MGA, Min-Min all increase too. This is mainly due to the fact that as the number of jobs increase, the system workload increases and results in the operation of Grid computing centers with low processing capacity. Therefore, more jobs get rejected as their execution finish times are beyond the deadline constraint. For low workloads, such as jobs that are 960, 1200, or 1440, there are only a few rejected jobs for the AJSM approach. However, as the number of jobs increases, the growth rate of the AJSM job rejection ratio is more than that of the MGA and Min-Min job rejection ratios. For high system workload, such as the number of jobs exceeds 2880, 5000, the job rejection ratio of AJSM may close to that of MGA. The main reason is that the deadline restriction becomes the key element of job rejection.

Fig 5(a) and 5(b) plot the job total processing time and average processing time of the three algorithms when the number of jobs increases from 960 to 2880. Fig 5(a) reveals that the AJSM job total processing time is more than that of MGA and Min-Min. This is a reasonable experimental phenomenon for AJSM handling more jobs, which results in a greater total processing time and lower job rejection ratio. This performance improvement manifests mainly in the average processing time of Fig 5(b), where AJSM exceeds MGA by 6.9% and Min-Min by 5.4%, for the average experimental results. The experimental results for comparison metric makespan are shown in Fig 5(c), where the AJSM outperforms MGA, Min-Min by an average of 6.2%, 5.4%, respectively. This is mainly due to the fact that our proposed ASJM strategy adopts two key techniques: job transmission time prediction based on the ARIMA model and heterogeneous Grid computing node resource normalization, which can give a more accurate job execution finish time. Therefore, our proposed ASJM is better than MGA, Min-Min in terms of average processing time, makespan, and job rejection ratio. From Fig 5, this paper can also conclude that Min-Min outperforms MGA in terms of average processing time and makespan, and MGA is better than Min-Min in term of job rejection ratio.

**Fig 6:**
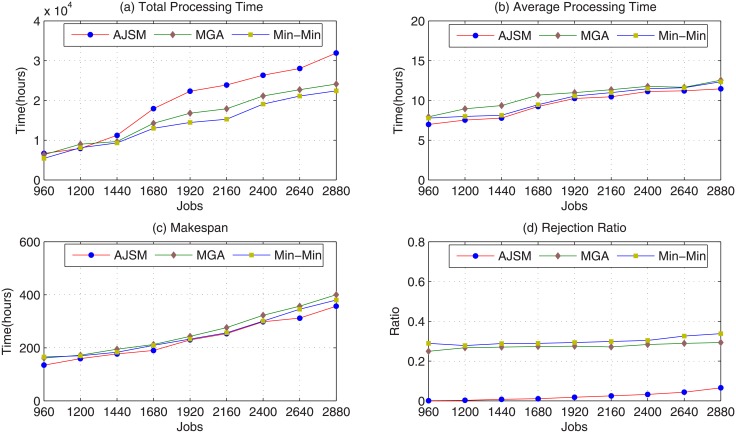
Performance impact of jobs with 120 scheduling points. (a) Total Processing Time; (b) Average Processing Time; (c) Makespan; (d) Job Rejection Ratio.

The improvements of AJSM over MGA and Min-Min could also be concluded from Fig 6, which shows the simulation experimental results with 120 scheduling periods. The AJSM algorithm significantly outperforms MGA by 91.6%, Min-Min by 92.3%, in term of job rejection ratio, respectively. Moreover, AJSM is also better than MGA by 10.7%, Min-Min by 5% in term of average processing time, and MGA by 9.7%, Min-Min by 5.8% in term of makespan. On the other hand, the average processing time and job rejection ratio of AJSM algorithm are superior to those of the experimental results with 60 scheduling periods. This is mainly due to the fact that the Grid systems’ workload with 120 scheduling periods is lower than the workload with 60 scheduling periods, and the AJSM can find a more optimal Grid computing center with the minimum execution finish time.

## Conclusions and future work

In this paper, our main objective was to effectively deal with Grid application software, hardware, and deadline requirements. Therefore, this paper first built a Grid job scheduling architecture that can periodically make job scheduling decisions. This paper then used an ARIMA model to forecast job transmission times. Next, this paper normalized the Grids’ heterogeneous computing nodes and formulated the application-aware deadline constraint job scheduling problem as a linear programming problem. Lastly, an AJSM scheduling mechanism was proposed to solve this problem with low time complexity. The comparison studies demonstrated that our proposed AJSM can successful schedule more Grid jobs than MGA, Min-Min. This is mainly due to the fact that the AJSM algorithm has a lower job rejection ratio than MGA and Min-Min. For successful scheduled jobs, AJSM scheduling mechanism also outperforms existing algorithms: MGA, Min-Min in terms of job average processing time and makespan.

Future studies in this area are twofold. First, we shall extend the Grid job transmission time prediction using an artificial neural network. Second, we plan to build a more precise job requirements model to describe Grid applications.

## Supporting information

S1 File Experimental results dataset S1_File.docx. (DOCX)

## References

[1] Haider S, Nazir B. Dynamic and Adaptive Fault Tolerant Scheduling With QoS Consideration in Computational Grid. IEEE Access 2017 Apr; 5: 7853–7873.

[2] Bellavista P, Cinque M, Corradi A, Foschini L, Frattini F, Povedano-Molina J. GAMESH: A grid architecture for scalable monitoring and enhanced dependable job scheduling. Future Gener. Comput. Syst. 2017 6;71:192–201. 10.1016/j.future.2016.10.023

[3] Tang XY, Li KL, Qiu MK, Sha EHM. A Hierarchical Reliability-Driven Scheduling Algorithm in Grid Systems. J. Parallel and Distributed Computing 2012 4;72(4):525–535. 10.1016/j.jpdc.2011.12.004

[4] http://www.cngrid.org/, Accessed 20 Apr. 2018.

[5] Abraham GT, James A, Yaacob N. Group-based Parallel Multi-scheduler for Grid computing. Future Gener. Comput. Syst. 2015 9;50:140–153. 10.1016/j.future.2015.01.012

[6] Dong F, Akl SG. Scheduling algorithms for grid computing: State of the art and open problems. Technical Report 2006; 504.

[7] Tiwari PK, Vidyarthi DP. Improved auto control ant colony optimization using lazy ant approach for grid scheduling problem. Future Gener. Comput. Syst. 2016 1;60:78–89. 10.1016/j.future.2016.01.017

[8] Tang XY, Liao XY, Zheng J, Yang XP. Energy Efficient Job Scheduling with Workload Prediction on Cloud Data Center. Cluster Computing 2018 9;21(3):1581–1593. 10.1007/s10586-018-2154-7

[9] http://www.cngrid.org/yxqk/qtjk/, Accessed 20 Apr. 2018.

[10] Hu M, Veeravalli B. Requirement-Aware Scheduling of Bag-of-Tasks Applications on Grids with Dynamic Resilience. IEEE Trans. computers, 2013 10;62(10):2108–2114. 10.1109/TC.2012.164

[11] https://altairhyperworks.com/product/RADIOSS, Accessed 12 Apr. 2018.

[12] Chen DY, Wang LM, Wang CZ, Yuan LK, Zhang TY, Zhang ZZ. Finite element based improvement of a light truck design to optimize crashworthiness. International J. Automotive Technology 2015 1;16(1):39–49. 10.1007/s12239-015-0004-7

[13] Tang JJ, Zhang S, Chen XQ, Fang Liu, Zou YJ. Taxi Trips Distribution Modeling Based on Entropy-maximizing Theory: A Case Study in Harbin City-China. Physica A: Statistical Mechanics and its Applications 2018 3;493:430–443. 10.1016/j.physa.2017.11.114

[14] Tang XY, Li XC, Fu ZJ. Budget-constraint Stochastic Task Scheduling on Heterogeneous Cloud Systems. Concurr. Comput.: Pract. Exper. 2017 10;29(19):1–13. 10.1002/cpe.4210

[15] Vaaheedha KS, Nazreen BM. MiM-MaM: A new task scheduling algorithm for grid environment. 2015 International Conference on Advances in Computer Engineering and Applications 2015; 695–699.

[16] Kołodziej J, Xhafa F. Enhancing the genetic-based scheduling in computational grids by a structured hierarchical population. Future Gener. Comput. Syst. 2011 8;27(8):1035–1046. 10.1016/j.future.2011.04.011

[17] Prakash S, Vidyarthi DP. Maximizing availability for task scheduling in computational grid using genetic algorithm. Concurr. Comput.: Pract. Exper. 2015 1;27(1):193–210. 10.1002/cpe.3216

[18] Younis MT, Yang S. A genetic algorithm for independent job scheduling in grid computing. MENDEL Soft Comput. J. 2017 1;23(1):65–72.

[19] Tiwari PK, Vidyarthi DP. Observing the effect of interprocess communication in auto controlled ant colony optimization-based scheduling on computational grid. Concurr. Comput.: Pract. Exper. 2014 1;26(1):241–270. 10.1002/cpe.2977

[20] Wang Y, Ma XL, Liu MW, Gong K, Liu Y, Xu MZ, Wang YH. Cooperation and profit allocation in two-echelon logistics joint distribution network optimization. Applied Soft Computing 2017 7;56:143–219. 10.1016/j.asoc.2017.02.025

[21] Liu H, Abraham A, Hassanien AE. Scheduling job on computational grids using a fuzzy particle swarm optimization algorithm. Future Gener. Comput. Syst. 2010 10;26(8):1336–1343. 10.1016/j.future.2009.05.022

[22] Tang JJ, Yang YF, Qi Y. A hybrid algorithm for urban transit schedule optimization. Physica A: Statistical Mechanics and its Applications 2018 12;512:745–755. 10.1016/j.physa.2018.08.017

[23] Abouelela M, El-Darieby M. Scheduling big data applications within advance reservation framework in optical grids. Applied Soft Computing 2016 1;38:1049–1059. 10.1016/j.asoc.2015.08.032

[24] Xu H, Yang B. An incentive-based heuristic job scheduling algorithm for utility grids. Future Gener. Comput. Syst. 2015 1; 49:1–7. 10.1016/j.future.2015.02.002

[25] Duwairi R, Abu-Rahmeh M. A novel approach for initializing the spherical K-means clustering algorithm. Simulation Modelling Practice and Theory 2015;54:49–63. 10.1016/j.simpat.2015.03.007

[26] Abrishami S, Naghibzadeh M, Epema D. Cost-Driven Scheduling of Grid Workflows Using Partial Critical Paths. IEEE Trans. Parallel and Distributed System 2012 8;23(8):1400–1414. 10.1109/TPDS.2011.303

[27] Wang Y, Ma XL, Lao YT, Wang YH. A fuzzy-based customer clustering approach with hierarchical structure for logistics network optimization. Expert Systems with Applications 2014 2;41(2,1):521–534. 10.1016/j.eswa.2013.07.078

[28] Wang Y, Ma XL, Li ZB, Liu Y, Xu MZ, Wang YH. Profit distribution in collaborative multiple centers vehicle routing problem. J. of Cleaner Production 2017 2;144:203–219. 10.1016/j.jclepro.2017.01.001

[29] Xhafa F, Carretero J, Barolli L, Durresi A. Immediate mode scheduling in grid systems. Int. J. Web Grid Serv. 2017 2;3(2):219–236. 10.1504/IJWGS.2007.014075

[30] Abdoli M, Entezari-Maleki R, Movaghar A. A rank-Based hybrid algorithm for scheduling data- and computation-Intensive jobs in grid environment in:Intelligent Computing, Networking, and Informatics, Springer, India, 2014;785–796.

[31] https://www.top500.org/lists/2017/11/, Accessed 15 Apr. 2018.

[32] Lin Y, Wang F, Liu B. Random number generators for large-scale parallel Monte Carlo simulations on FPGA. J. Computational Physics 2018;360:93–103. 10.1016/j.jcp.2018.01.029

[33] Zhou H, Li P, Xie W, Hussain S, He Y. Genome-wide Association Analyses Reveal the Genetic Basis of Stigma Exsertion in Rice. Molecular Plant 2017 4;10(4):634–644. 10.1016/j.molp.2017.01.001 28110091 10.1016/j.molp.2017.01.001

[34] Box G, Jenkins G, Reinsel G. Time Series Analysis, Forecasting and Control. third ed, Prentice-Hall; 1994.

[35] Zhang G, Zhu X, Bao W, Yan H, Tan D. Local Storage-Based Consolidation With Resource Demand Prediction and Live Migration in Clouds. IEEE Access 2018;6:26854–26865. 10.1109/ACCESS.2018.2825354

[36] Tang JJ, Liang J, Zhang S, Huang HL, Fang Liu. Inferring driving trajectories based on probabilistic model from large scale taxi GPS data. Physica A: Statistical Mechanics and its Applications 2018 9;506:566–577. 10.1016/j.physa.2018.04.073

[37] Qiao WT, Wang J, Song MX, Wen Y. Wind farm micro-siting based on auto-regressive wind prediction. The 2015 IEEE Conference on Control Applications (CCA) 2014; 1853–1855.

[38] http://www.nscc-gz.cn/Product/HighPerformanceComputingService/ServiceCharacteristics.html, Accessed 21 Apr. 2018.

[39] http://www.nsccsz.gov.cn/hpc/resources/hardwares, Accessed 22 Apr. 2018.

[40] Zou YJ, John E, Park BJ, Lord D, Wu LG. Empirical Bayes estimates of finite mixture of negative binomial regression models and its application to highway safety. Journal of Applied Statistics 2018 9;45(9):1652–1669. 10.1080/02664763.2017.1389863

[41] Fan SS, Tian H, Wang WD. A Radio Resource Virtualization-Based RAT Selection Scheme in Heterogeneous Networks. IEEE Communications Letters 2017 5;21(5):1147–1150. 10.1109/LCOMM.2017.2664808

[42] Hao Y, Liu G, Wen N. An enhanced load balancing mechanism based on deadline control on GridSim. Future Gener. Comput. Syst. 2012 4;48(4):657–665. 10.1016/j.future.2011.10.010

[43] ParallelWorkloads Archive. Available from: http://www.cs.huji.ac.il/labs/parallel/workload/. Accessed April 12, 2018.

