# Supplementary material for: Application-aware deadline constraint job scheduling mechanism on large-scale computational grid
Source: PLoS One. 2018 Nov 20;13(11):e0207596. doi: 10.1371/journal.pone.0207596 (PMC6245787; doi:10.1371/journal.pone.0207596)
Supplement: S1 File — (DOCX) [file pone.0207596.s001.docx]

Supporting Information data

Periodic 60，4hour

960 1200 1440 1680 1920 2160 2400 2640 2880

A1=[6756 8148 11736 16614 19986 18900 22416 21162 23928];

B1=[7.03 6.79 8.15 10.26 10.74 10.5 12.1 11.01 11.7];

C1=[0.001 0.0034 0.012 0.0183 0.02094 0.03167 0.0425 0.0732 0.1529];

A2=[4838 6970 8635 11899 13724 14355 16304 17974 19089];

B2=[7.73 8.28 8.35 10.66 10.374 11.5 11.8 12.01 12.57];

C2=[0.329 0.331 0.332 0.356 0.362 0.367 0.389 0.388 0.395];

A3=[5322 7921 9334 12199 14544 15155 17475 18682 21570];

B3=[7.43 7.79 8.23 10.46 10.94 11.2 11.6 11.89 12.2];

C3=[0.287 0.288 0.29 0.291 0.312 0.326 0.339 0.357 0.378];

Periodic 120，8hour

D1=[6556 7948 11236 17814 21986 23900 26416 28162 31928];

E1=[6.93 7.12 7.85 9.36 10.24 10.45 11.12 11.2 11.45];

F1=[0.001 0.0025 0.0078 0.011 0.018 0.0247 0.0325 0.0432 0.06529];

D2=[4938 6770 8835 11499 12724 14455 16904 18674 20889];

E2=[7.83 8.14 8.75 9.66 10.784 11.15 11.78 11.93 12.37];

F2=[0.319 0.341 0.339 0.348 0.357 0.359 0.377 0.367 0.385];

D3=[5222 7901 9304 12899 14944 14955 17675 18382 21270];

E3=[7.33 7.59 8.13 9.46 10.54 10.98 11.46 11.83 12.12];

F3=[0.289 0.278 0.2879 0.28891 0.29312 0.298326 0.3039 0.3257 0.3378];
